# Review on porcine endogenous retrovirus detection assays—impact on quality and safety of xenotransplants

**DOI:** 10.1111/xen.12154

**Published:** 2015-01-31

**Authors:** Antonia W Godehardt, Michael Rodrigues Costa, Ralf R Tönjes

**Affiliations:** Division of Medical Biotechnology, Paul-Ehrlich-InstitutLangen, Germany

**Keywords:** cocultivation, microarray analysis, porcine endogenous retrovirus, RNA-Seq, xenotransplantation

## Abstract

Xenotransplantation of porcine organs, tissues, and cells inherits a risk for xenozoonotic infections. Viable tissues and cells intended for transplantation have to be considered as potentially contaminated non-sterile products. The demands on microbial testing, based on the regulatory requirements, are often challenging due to a restricted shelf life or the complexity of the product itself. In Europe, the regulatory framework for xenogeneic cell therapy is based on the advanced therapy medicinal products (ATMP) regulation (2007), the EMA CHMP Guideline on xenogeneic cell-based medicinal products (2009), as well as the WHO and Council of Europe recommendations. In the USA, FDA guidance for industry (2003) regulates the use of xenotransplants. To comply with the regulations, validated test methods need to be established that reveal the microbial status of a transplant within its given shelf life, complemented by strictly defined action alert limits and supported by breeding in specific pathogen-free (SPF) facilities. In this review, we focus on assays for the detection of the porcine endogenous retroviruses PERV-A/-B/-C, which exhibit highly polymorphic proviral loci in pig genomes. PERVs are transmitted vertically and cannot be completely eliminated by breeding or gene knock out technology. PERVs entail a public health concern that will persist even if no evidence of PERV infection of xenotransplant recipients in vivo has been revealed yet. Nevertheless, infectious risks must be minimized by full assessment of pigs as donors by combining different molecular screening assays for sensitive and specific detection as well as a functional analysis of the infectivity of PERV including an adequate monitoring of recipients.

## Introduction

For xenotransplantation, pigs show numerous advantages as donor animals compared with other choices such as non-human primates, which have been established vice versa as recipient animals for preclinical pig organ xenotransplantation [1,2]. In addition to the anatomical, immunological, and physiological barriers, the risk of infection for the recipient needs to be clarified. Valid and broad-range detection assays for donor and manufacture derived microorganisms are the method of choice and must be established. These could include microarrays that have already proven their suitability for the screening of retroviruses [3] and/or next-generation sequencing (NGS)-based methods [4,5].

Besides the general microbial detection, the selection of appropriate donor pigs reveals a crucial step. For endogenous retroviruses, this includes the screening for the absence of PERV-C proviruses in the pig genome, as genetic recombinants between PERV-A and PERV-C show high replication titers [6]. Recombination events of this quality should be generally avoided. The pigs should demonstrate low or even no expression of PERV-A, PERV-B as well as PERV-C as the impact of PERV-A/C recombinants on the health of xenotransplant recipients cannot be fully estimated. They might be exposed to a lifelong challenge with the virus. If putative PERV-C-positive animals are considered as donors for transplantation as performed [7], the quality of PERV-C sequences, if present in donor animals, should be evaluated for *gag*, *pol* (*prt*, *int*, RT), *env* open reading frames, and LTR structures. Functional PERV-C needs to be distinguished from non-functional provirus. Several approaches are established for quality control and characterization of PERVs infective potential.

To differentiate between pigs with low and high expression of PERV, assays were developed that are based on mitogen stimulation of PBMCs [8].

Other currently used and well-known methods to detect and analyze the presence of PERV focus on direct detection of (i) provirus in the cells, (ii) the expression analysis of viral mRNA, (iii) the detection of viral proteins or (iv) the production of infectious viruses itself. The nucleic acid methodology that has been considered as valid testing method also for clinical trials is based on PCR and real-time PCR methods. Southern blot hybridization using PERV-specific primers and probes, melting assays to quantify PERV copy numbers, as well as fluorescence in situ hybridization (FISH) for chromosomal localization are additional alternatives. The measurement of viral reverse transcriptase activity (RT test) indicates virus production. Indirect detection of PERV is carried out by analyzing the recipient's antibody immune response. This is mainly based on ELISA, Western blot analysis testing the recipient's sera with purified virus, recombinant protein, or synthetic peptides [8,9].

Vaccination of human recipients as a strategy to prevent PERV transmission represents a theoretical choice. An approach by induction of neutralizing antibodies has been suggested [10].

A successful inhibition of PERV expression in vitro was achieved by small interfering RNAs (siRNAs) specific for the PERV *pol* gene [11]. A lentiviral vector expressing a short hairpin RNA (shRNA) of PERV *pol* led to downregulation of PERV expression in vitro [12]. Similarly, pigs transgenic for the PERV-specific shRNA showed significantly inhibited PERV expression in different tissues [13]. Analogous results were obtained using siRNAs in vivo [14]. The targeted knockout of PERV, for example, by the application of zinc-finger nucleases for the generation of genetically modified animals is complicated due to the large number of proviruses The absence of functional PERV-C as well as the selection of low-producer animals (PERV-A/-B) should be a demand absolute requirement for the generation of donor pigs intended for xenotransplantation.

Approaches to achieve PERV knockouts using zinc-finger nucleases have failed. No viable offspring has been obtained. However, other systems such as Sleeping Beauty or CRISPR/Cas technology are in place that may show better performance [15,16]. Nevertheless, as the presence of non-functional PERV relicts does not affect the quality of xenotransplants, pigs free of functional PERV-C should be used as founder animals for breeding. Their offspring should be chosen as donor animals for xenotransplantation.

The expected increase in xenotransplantation events when starting first in man trials will possibly lead to marketing authorized medicinal products. As such, xenotransplants could be placed on the market. Even if no patient was affected yet, it does not necessarily mean that PERV does not have the potential for being infectious in vivo either in susceptible and/or immunosuppressed patients. In addition, PERV transmission in different human cell lines was repeatedly demonstrated in vitro in the past, which shows that a putative potential risk may exist and should not be ignored. For this reason, pigs have to be selected carefully to exclude the slightest risk. At any rate, the generation of pigs without expression of functional PERV remains the major goal given that the absence of the retrovirus is prevention at its best.

## Update on PERV detection assays

### Diagnostic detection of PERV transmission via cocultivation

A sensitive and established test for PERV transmission is the cocultivation of PERV producer cells and human target cells. In practice, virus-producing cells were exposed to a certain, lethal irradiation dose so that producer cells died a few days post-cocultivation. The irradiation dose depended on the source and had to be adjusted individually in a pre-experimental setting [17]. Putatively infected cells were exclusively cultivated until monitoring time had expired, usually after 4–8 weeks. By means of this system, discrimination between PERV releasing cells and PERV target cells was guaranteed to the greatest possible extent [18,19]. Based on this strategy, activated porcine peripheral blood mononuclear cells (poPBMC) were cocultivated with human embryonic kidney (HEK) 293 cells [17]. The results obtained were of great importance. The capacity of PERV-C to be recombined with PERV-A in vitro was demonstrated for the first time. Furthermore, the assay revealed a chance to analyze the infectious potential of functional PERV as well as the susceptibility of certain cell lines for PERV. The newly recombined PERV-A/C infected human cell lines ex vivo at titers higher than those observed for parental PERV particles. In addition, outcomes of the same study revealed that PERV-A/C expressed in producer 293/PERV-NIH-3° cells just as poPBMC fail to infect human PBMC (huPBMC) in cocultures [17]. This indicated that huPBMC express a solid innate immunological barrier apparently counter-regulating PERV during infection. The cocultivation of gamma-irradiated virus-producing cells with target human cells has become a common approach [6,20–22]. Nevertheless, this methodology bears a great disadvantage. The cells are not strictly separated, and the only method to eliminate the PERV source is to irradiate the producer cells. This scenario significantly deviates from the real situation of pig-to-human xenotransplantation, and it may affect the characteristics of PERV as well.

In the newly developed cocultivation strategies, the implementation of a two chamber system was a significant improvement [18,19]. This system is based on target cells that are seeded, for example, in the bottom of 6-well plates combined with upper hanging cell culture inserts containing the producer cells. The PERV producer cells are separated from pre-seeded target cells by a porous membrane [18,19]. The pore size of 0.4 *μ*m allows microcirculation of small particles and viruses through the membrane resulting in intercellular communication as well as receptor-mediated virus infection. However, cell migration into the subsection with target cells is impeded and previously observed side effects such as microchimerism are avoided. This assay offers two possibilities, first to investigate PERV infectious potential and second to differentiate PERV-susceptible from non-susceptible cells. This in vitro scenario mimics the in vivo pig-to-human xenotransplantation event as closely as possible given that both cell types (*virus donor* and *virus target)* were maintained viable during the entire cocultivation experiment [19]. The intracellular communication via small molecules that is enabled by virtue of the cocultivation strategy opens new avenues to explore immunological or further virological aspects involved in PERV infection and counteraction in target cells.

The feasibility of this approach was demonstrated successfully in different studies that were aimed to address PERV infectious potential. Cocultivation of 293 cells producing either the PERV-A/C recombinant PERV/5° or PERV-B with mouse 3T6 cells resulted in a non-productive infection of mouse cells as no PERV provirus was detected in 3T6 cells by PCR specific for each PERV class [23]. Another study described the non-capacity of PERV expressed by mitogenically activated poPBMC purified from Göttingen minipigs to infect permissive human 293 cells via cocultivation. Except for positive control experiments, which involved the cocultivation of PERV/5°-producing 293 cells with naive 293 cells, no provirus was detected in target 293 cells [18].

The advantage of cocultivation without preceding irradiation is obvious. Both cell types are maintained viable during the complete experimental time, and irradiation of primary virus source was not required. Virus particles are able to diffuse through the membrane and infect susceptible target cells without any risk of microchimerism. Due to these options, the coculture technique could be introduced as a new standard. It may be used to select animals for cloning that have revealed no transmission of PERV. The testing and selection of parents being negative for transmission after coculture experiments could be the basis for the generation of transmission-negative donor animals.

### Quantitative and differential gene expression profiling by microarray analysis

Besides the other well established molecular screening methods [19,24,25] microarray technology evolved as a powerful diagnostic tool. It is suitable for multiplex, selected detection, and characterization of microorganisms including bacteria, viruses, fungi, or parasites in patient samples and/or medicinal products intended for human application [26–32]. The scope for microarray analyses is widespread reaching from peptide or protein arrays via RNA—to dsDNA—or ssDNA arrays as well as Exon- and miRNA microarrays that are available, for example, for custom gene expression and species-specific genome analysis. They basically follow the same principle. Distinct *probes* are spotted as discrete features on a solid surface, commonly glass slides and hybridized, for example, with a fluorescent cyanine dye labeled sample of interest. This sample contains the so-called *target* sequences, which should exclusively hybridize with its complementary probes. Detection occurs by measurement of the fluorescent intensity with an, for example, specific laser-induced fluorescence scanner, counting all features that exceed the application-dependent background limit according to internationally specified guidance values [33]. The DNA microarray techniques available for gene expression profiling or analysis of genomic DNA among others range from printed DNA microarrays, in situ-synthesized oligonucleotide microarrays, suspension bead arrays to high-density bead arrays. The number of features ranges from less than a hundred (low-density arrays) up to 1 million (high-density arrays). Commercially available systems hereby provide a broad range of different preselected collections as well as custom designs. According to probe design and application, microarrays vary in their specificity and sensitivity for the expected target by assigning short or long probe sequences ranging, for example, from 20 nucleotides (20-mer) up to 150 nucleotides (150-mer) that are directed against a unique sequence of the corresponding target gene [34–36]. In this case, DNA quality is the crucial factor as any mismatch has a dramatic impact on the performance of oligonucleotides while influencing the stability of duplex formation and its great dynamic range. Another important factor is the level of signal-to-noise ratio that is most relevant to achieve significant results. According to Hughes et al., the absolute detection limit of 60-mer oligonucleotide consisting ink-jet arrays is close to 0.1 copies/cell equivalent, or 1 : 1 000 000, based on 100 000 transcripts/cell [37–40].

As microarray technology has already been successfully used for retroviral/viral screening including human endogenous retroviruses (HERV) or exogenous retroviruses such as HIV or HTLV [3], avian influenza virus [29], coxsackie, and other enteroviruses [26,27] as well as applications in clinical diagnosis [38], it is obvious to manifest this method as a PERV detection assay to support routine analysis of quality and safety of porcine-derived products.

In a recent approach, highly specific porcine diagnostic gene probes were spotted in a customized design (*MyArray*; OakLabs GmbH, Hennigsdorf, Germany) as 8x60K arrays [41]. Probe data were derived from the annotated complete draft pig genome sequence, published by the *Swine Genome Sequencing Consortium* in November 2012 [42]. These data comprise relevant porcine inflammatory and host restriction factors amended by conserved regions of PERV-A/-B/-C *env* and *prt/pol* and selected human transgenes. The data may reveal comprehensive information on the retroviral status as well as on tissues viability and quality that far exceeds the properties of multiplex PCR or RT-PCR that are solely used for affirmative analysis.

In summary, microarray technology in combination with quantitative RT-PCR, for example, for selected genes allows cost-efficient testing of samples within the given and often restricted shelf life of the product. It does reveal the specific PERV status on the one hand and provides broad information on differential gene expression profiles on the other hand. This fast method is suitable for parallel testing of different samples derived from one or several putative donor animals. Furthermore, it may reveal the effect of gene transfer/knockout on the expression profile of selected target cell lines that are potentially intended as ATMP.

### RNA-Seq

In addition to microarray analyses, genome technologies provide another meaningful tool for pathogen detection and gene expression analysis. Deep sequencing technologies such as RNA-Seq are intended to enable precise measurement of levels of transcripts and their isoforms not restricted to a predetermined selection of probes for particular targets. By maintaining a sufficient reading depth and read length, RNA-Seq is a well-suited detection method for qualitative expression profiling, generally comparable with microarray analysis, particularly for organisms encompassing unknown genomic sequence targets [4,43–45]. As such, RNA-Seq constitutes an indispensable method for foreign pathogen detection, PERV expression profiling, and subsequent transcriptome analysis. It is sufficient for the selection of suitable animals intended for xenotransplantation or screening of qualified animals that are intended for further pathogen-free breeding. As it is not limited to a certain number of gene targets, it offers the chance to identify novel candidate genes and gene polymorphisms as shown in recent studies on boar testis and liver tissues as well as in other reference organisms [46–48].

The issues of microarrays and deep sequencing are new for pigs in general. They are considered as general assays capable of covering PERV-specific issues such as expression levels as well as the effect(s) on cells looking into the differential gene expression profiles. In addition to the microbial safety aspect, both assays provide a broad insight into the expression status of cells, for example, for comparative analysis of native and genetically modified cells and cell lines.

## Regulatory requirements

In addition to the scientific approaches, biological medicinal products intended for placing on the market require marketing authorizations, based on the regulatory requirements of the appropriate national agencies and competent authorities in conformity with their legal frameworks. In Europe, xenogeneic products are subject to the regulations of ATMPs, which are established in Regulation (EC) No 1394/2007 [49]. The European Medicines Agency (EMA) provides the necessary information for applicants on classification and certification of the quality and non-clinical data including support to companies and guidance on the valid regulatory framework. Furthermore, for xenogeneic cell-based medicinal products that are dedicated to the field of cell therapy and tissue engineering, specific regulatory information is provided in the CHMP Guideline on xenogeneic cell-based medicinal products (EMEA/CHMP/CPWP/83508/2009). The guideline on xenogeneic cell-based medicinal products [50] addresses the minimum requirements regarding quality and manufacturing aspects including testing. It has a focus on source animals, their procurement, and processing in GMP-certified manufacturing facilities. In particular, the surveillance of known and unknown infectious agents in source and founder animals that are kept under SPF conditions, with adequate and validated diagnostic assays is the basis for appropriate quality assurance. Advice on microbial testing methods and their validation is among other sources given in the European Pharmacopoeia, Ph. Eur. 5.1; 5.1.6; 2.6.1, and 2.6.27. The methods should be well defined and should follow appropriate laboratory assurance standards. For pigs as source animals, besides zoonotic, human pathogenic microbial agents, special consideration is given to the screening of porcine endogenous retroviruses (PERV). It is advised to apply methods such as hybridization, antibody testing, and/or PCR as well as classical methods such as pathology and histopathology. The relevant CHMP guidelines for clinical trials, including the guideline on human cell-based medicinal products (EMEA/CHMP/410896/2006) [51], should be taken into account as recommended in EMEA/CHMP/CPWP/83508/2009 [50]. Nevertheless, the evaluation of a clinical trial including its safety and risk evaluation especially in the field of ATMPs requires a product-specific assessment process, which depends on the current legal framework as well as on the growing knowledge on advanced therapy medicinal products. Further information is provided, for example, by European Parliament, WHO and Council of Europe recommendations [51–57].

In the USA, the use of xenotransplantation products in humans is regulated by the Food and Drug Administration (FDA) as the competent authority. A regulatory framework is provided by the *Guidance for Industry* documents published in 2002 and 2003 [58,59].

## Outlook

The presented update on assays for PERV detection and gene expression analysis displays a wide diversity of testing methods that support the generation of pigs as donors of tissues and cells to fulfill the regulatory prerequisites on safety and quality according to the international regulatory requirements for xenotransplantation clinical trials [60]. The choice of methods strongly depends on the target materials and needs adaptation to each approach. The selection of PERV-C free, PERV-A/-B low-producer animals in addition to the full assessment of the microbial background including potential zoonotic microorganisms and environmental microorganisms incorporated during processing is indispensable [8]. Methods on cocultivation and gene expression profiling offer new approaches to generate data that help to evaluate the risk/benefit balance of the individual product to provide safe xenotransplants in the future [61].

## Abbreviations

ATMP: Advanced therapy medicinal product
CHMP: Committee for Medicinal Products for Human Use
EMA: European Medicines Agency
FDA: Food and Drug Administration
GMP: Good Manufacturing Practice
huPBMC: human peripheral blood mononuclear cells
NGS: Next-generation sequencing
PERV: Porcine endogenous retrovirus
Ph. Eur.: European Pharmacopoeia
poPBMC: porcine peripheral blood mononuclear cells
PCR: Polymerase chain reaction
RT-PCR: Reverse-transcriptase polymerase chain reaction
SPF: specific pathogen-free
WHO: World Health Organization
XTx: Xenotransplantation

## References

[1] Manji RA, Ekser B, Menkis AH, Cooper DK (2014). Bioprosthetic heart valves of the future. Xenotransplantation.

[2] Zhou H, Iwase H, Wolf RF (2014). Are there advantages in the use of specific pathogen-free baboons in pig organ xenotransplantation models?. Xenotransplantation.

[3] Seifarth W, Spiess B, Zeilfelder U (2003). Assessment of retroviral activity using a universal retrovirus chip. J Virol Methods.

[4] Wang Z, Gerstein M, Snyder M (2009). RNA-Seq: a revolutionary tool for transcriptomics. Nat Rev Genet.

[5] Zou W, Chen D, Xiong M (2013). Insights into the increasing virulence of the swine-origin pandemic H1N1/2009 influenza virus. Sci Rep.

[6] Wilson CA, Wong S, Muller J (1998). Type C retrovirus released from porcine primary peripheral blood mononuclear cells infects human cells. J Virol.

[7] Wynyard S, Nathu D, Garkavenko O, Denner J, Elliott R (2014). Microbiological safety of the first clinical pig islet xenotransplantation trial in New Zealand. Xenotransplantation.

[8] Denner J, Tönjes RR (2012). Infection barriers to successful xenotransplantation focusing on porcine endogenous retroviruses. Clin Microbiol Rev.

[9] Kimsa MC, Strzalka-Mrozik B, Kimsa MW (2014). Porcine endogenous retroviruses in xenotransplantation-molecular aspects. Viruses.

[10] Waechter A, Denner J (2014). Novel neutralising antibodies targeting the N-terminal helical region of the transmembrane envelope protein p15E of the porcine endogenous retrovirus (PERV). Immunol Res.

[11] Karlas A, Kurth R, Denner J (2004). Inhibition of porcine endogenous retroviruses by RNA interference: increasing the safety of xenotransplantation. Virology.

[12] Dieckhoff B, Karlas A, Hofmann A (2006). Inhibition of porcine endogenous retroviruses (PERVs) in primary porcine cells by RNA interference using lentiviral vectors. Arch Virol.

[13] Dieckhoff B, Petersen B, Kues WA (2008). Knockdown of porcine endogenous retrovirus (PERV) expression by PERV-specific shRNA in transgenic pigs. Xenotransplantation.

[14] Ramsoondar J, Vaught T, Ball S (2009). Production of transgenic pigs that express porcine endogenous retrovirus small interfering RNAs. Xenotransplantation.

[15] Ivics Z, Garrels W, Mátés L (2014). Germline transgenesis in pigs by cytoplasmic microinjection of Sleeping Beauty transposons. Nat Protoc.

[16] Hai T, Teng F, Guo R, Li W, Zhou Q (2014). One-step generation of knockout pigs by zygote injection of CRISPR/Cas system. Cell Res.

[17] Wilson CA, Wong S, Vanbrocklin M, Federspiel MJ (2000). Extended analysis of the in vitro tropism of porcine endogenous retrovirus. J Virol.

[18] Semaan M, Rotem A, Barkai U, Bornstein S, Denner J (2013). Screening pigs for xenotransplantation: prevalence and expression of porcine endogenous retroviruses in Göttingen Minipigs. Xenotransplantation.

[19] Rodrigues Costa M, Fischer N, Gulich B, Tönjes RR (2014). Comparison of porcine endogenous retroviruses infectious potential in supernatants of producer cells and in cocultures. Xenotransplantation.

[20] Patience C, Takeuchi Y, Weiss RA (1997). Infection of human cells by an endogenous retrovirus of pigs. Nat Med.

[21] Ericsson TA, Takeuchi Y, Templin C (2003). Identification of receptors for pig endogenous retrovirus. Proc Natl Acad Sci USA.

[22] Garkavenko O, Wynyard S, Nathu D (2008). Porcine endogenous retrovirus transmission characteristics from a designated pathogen-free herd. Transplant Proc.

[23] Irgang M, Karlas A, Laue C (2005). Porcine endogenous retroviruses PERV-A and PERV-B infect neither mouse cells in vitro nor SCID mice in vivo. Intervirology.

[24] Kaulitz D, Mihica D, Dorna J (2011). Development of sensitive methods for detection of porcine endogenous retrovirus-C (PERV-C) in the genome of pigs. J Virol Methods.

[25] Wynyard S, Garkavenko O, Elliot R (2011). Multiplex high resolution melting assay for estimation of Porcine Endogenous Retrovirus (PERV) relative gene dosage in pigs and detection of PERV infection in xenograft recipients. J Virol Methods.

[26] Wang D, Coscoy L, Zylberberg M (2002). Microarray-based detection and genotyping of viral pathogens. Proc Natl Acad Sci USA.

[27] Wang D, Urisman A, Liu YT (2003). Viral discovery and sequence recovery using DNA microarrays. PLoS Biol.

[28] Lin B, Blaney KM, Malanoski AP (2007). Using a resequencing microarray as a multiple respiratory pathogen detection assay. J Clin Microbiol.

[29] Lin B, Malanoski AP, Wang Z (2009). Universal detection and identification of avian influenza virus by use of resequencing microarrays. J Clin Microbiol.

[30] Hsiue HC, Huang YT, Kuo YL (2010). Rapid identification of fungal pathogens in positive blood cultures using oligonucleotide array hybridization. Clin Microbiol Infect.

[31] Ballarini A, Segata N, Huttenhower C, Jousson O (2013). Simultaneous quantification of multiple bacteria by the BactoChip microarray designed to target species-specific marker genes. PLoS One.

[32] Chen JX, Chen MX, Ai L (2012). A protein microarray for the rapid screening of patients suspected of infection with various food-borne helminthiases. PLoS Negl Trop Dis.

[33] Tenenbaum JD, Sansone SA, Haendel M (2014). A sea of standards for omics data: sink or swim?. J Am Med Inform Assoc.

[34] Miller MB, Tang YW (2009). Basic concepts of microarrays and potential applications in clinical microbiology. Clin Microbiol Rev.

[35] Wheelan SJ, Martínez Murillo F, Boeke JD (2008). The incredible shrinking world of DNA microarrays. Mol BioSyst.

[36] Stoughton RB (2005). Applications of DNA microarrays in biology. Annu Rev Biochem.

[37] Hughes TR, Mao M, Jones AR (2001). Expression profiling using microarrays fabricated by an ink-jet oligonucleotide synthesizer. Nat Biotechnol.

[38] Shi L, Reid LH, Jones WD, MAQC Consortium (2006). The MicroArray Quality Control (MAQC) project shows inter- and intraplatform reproducibility of gene expression measurements. Nat Biotechnol.

[39] Arezi B, Guha N, Bergstrom Lucas A, Agilent Technologies

[40] Leproust E, Agilent Technologies

[41] Godehardt AW, Tönjes RR (2013). Update on assays for detection of PERVs. Xenotransplantation.

[42] Groenen MA, Archibald AL, Uenishi H (2012). Analyses of pig genomes provide insight into porcine demography and evolution. Nature.

[43] Xu W, Seok J, Mindrinos MN (2011). Human transcriptome array for high-throughput clinical studies. Proc Natl Acad Sci USA.

[44] Robertson G, Schein J, Chiu R (2010). De novo assembly and analysis of RNA-Seq data. Nat Methods.

[45] Łabaj PP, Leparc GG, Linggi BE (2011). Characterization and improvement of RNA-Seq precision in quantitative transcript expression profiling. Bioinformatics.

[46] Gunawan A, Sahadevan S, Neuhoff C (2013a). RNA deep sequencing reveals novel candidate genes and polymorphisms in boar testis and liver tissues with divergent androstenone levels. PLoS One.

[47] Gunawan A, Sahadevan S, Cinar MU (2013b). Identification of the novel candidate genes and variants in boar liver tissues with divergent skatole levels using RNA deep sequencing. PLoS One.

[48] Hackl H, Burkard TR, Sturn A (2005). Molecular processes during fat cell development revealed by gene expression profiling and functional annotation. Genome Biol.

[49] Regulation (EC)

[50] EMEA/CHMP/CPWP/83508/2009, Committee for Medicinal Products for Human Use (CHMP) Guideline on Xenogeneic Cell-Based Medicinal Products.

[51] EMEA/CHMP/410896/2006, Committee for Medicinal Products for Human Use (CHMP) Guideline on Human Cell-Based Medicinal Products.

[52] EMA/CAT/CPWP/686637/2011, Committee for Advanced Therapies (CAT) Draft Guideline on the Risk-based Approach According to Annex I, part IV of Directive 2001/83/EC Applied to Advanced-therapy Medicinal Products.

[53] The Changsha Communiqué (2009). First WHO Global Consultation on Regulatory Requirements for Xenotransplantation Clinical Trials: Changsha, China, 19-21 November 2008. Xenotransplantation.

[55] Regulation (EC)

[56] Fishman JA, Scobie L, Takeuchi Y (2012). Xenotransplantation-associated infectious risk: a WHO consultation. Xenotransplantation.

[57] Cozzi E, Tallacchini M, Flanagan EB (2009). The International Xenotransplantation Association consensus statement on conditions for undertaking clinical trials of porcine islet products in type 1 diabetes–chapter 1: Key ethical requirements and progress toward the definition of an international regulato ry framework. Xenotransplantation.

[58] U.S. Department of Health and Human Services, Food and Drug Administration, Center for Biologics Evaluation and Research (CBER) (2002).

[59] U.S. Department of Health and Human Services, Food and Drug Administration, Center for Biologics Evaluation and Research (CBER) (2003).

[60] Noel L (2012). Global regulatory requirements for xenotransplantation clinical trials. Xenotransplantation.

[61] Tönjes RR (2013). Safe transplantation. Lifeline. Medical research for healthy lives. Int Innov.

